# UHPLC–MS Characterization, and Antioxidant and Nutritional Analysis of Cocoa Waste Flours from the Peruvian Amazon

**DOI:** 10.3390/antiox11030595

**Published:** 2022-03-21

**Authors:** Gabriel Vargas-Arana, Claudia Merino-Zegarra, Miguel Tang, Mariano Walter Pertino, Mario J. Simirgiotis

**Affiliations:** 1 Laboratorio de Química de Productos Naturales, Instituto de Investigaciones de la Amazonia Peruana, Av. Abelardo Quiñones, Iquitos 16001, Peru; cmerino@iiap.gob.pe; 2 Facultad de Industrias Alimentarias, Universidad Nacional de la Amazonía Peruana, Iquitos 16001, Peru; 3 Asociación Amazónicos por la Amazonía (AMPA), Mz. N Lote 1 Urb. Vista Alegre, Moyobamba 22001, Peru; tang_miguel@hotmail.com; 4 Laboratorio de Química de Productos Naturales, Instituto de Química de Recursos Naturales, Universidad de Talca, Casilla 747, Talca 3460000, Chile; mwalter@utalca.cl; 5 Instituto de Farmacia, Facultad de Ciencias, Campus Isla Teja, Universidad Austral de Chile, Valdivia 5090000, Chile

**Keywords:** antioxidant activity, cocoa flour, nutritional values, ESI–MS, phenolics

## Abstract

Cocoa (*Theobroma cacao)* is a food product used worldwide and a key raw material for chocolate manufacturing. Cocoa possesses bioactive compounds such as methylxanthines, flavonoids, procyanidins, and related molecules with medicinal or health-promoting properties. Cocoa shell and pod husk have been proposed as a by-product with several interesting bioactivities, and the gummy residue or glue (a sticky, gluey by-product known as “mucilage” in Spanish) is used to produce liquors and is eaten as a food in Perú. However, little is known about the chemical composition and bioactivity of flours made from Peruvian cocoa ecotype wastes such as those from the vein and pod husk of the fruits. This study aimed to characterize the in vitro antioxidant properties and nutritional values of flours made from the waste from a special ecotype of cocoa (CCN-51). The chemical fingerprinting was performed using UHPLC–HESI orbitrap mass spectrometry and allowed the detection of 51 compounds. GC-FID was used for the determination of individual fatty acid contents, and the antioxidant activity was assessed by several assays (DPPH, FRAP, and ABTS). The flours obtained were composed of a good amount of dietary fiber, carbohydrates, and minerals, as well as several bioactive polyphenolic compounds, fatty acids, and amino acids with nutraceutical properties, making the flours a rich and promising food as well as a good source for the preparation of functional foods or nutraceuticals.

## 1. Introduction

**Figure 1:**
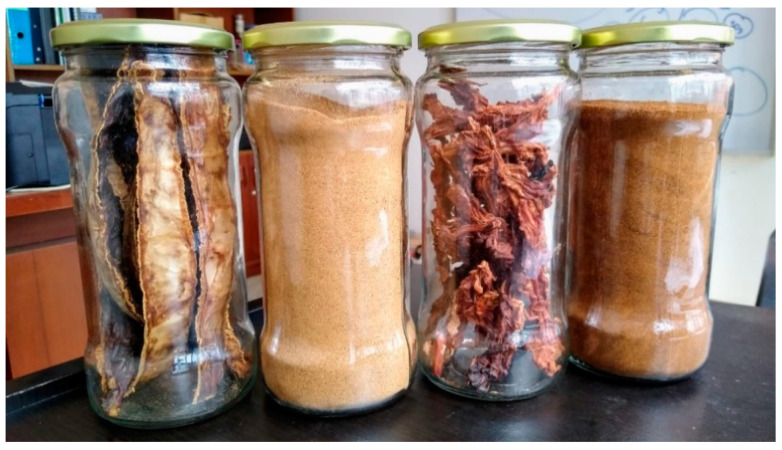
Pod husk and its flour (**left**) and vein and its flour (**right**).

In recent years, fruit and vegetable by-products or food wastes have been shown to be a good source of bioactive compounds that can be extracted and reintroduced into the food chain or in food matrices as natural food additives for the production of functional foods or nutraceuticals [1]. In addition, the reduction of food waste is growing as an important process of environmental and economical welfare. Cocoa by-products, primarily cocoa pod husks, are produced in vast quantities around the world, representing 70–80% of the dry weight of the fruit [2]. They are usually thrown away as a production waste, which has a detrimental environmental impact [3]. Cocoa beans are mainly used to produce cocoa powder and chocolate. It is estimated that a total of around 4–5 million tons of cocoa is produced worldwide per year [4] and 700,000 tons of cocoa bean shells are produced as waste every year [1], being a good source of proteins, fatty acids, and reported polyphenolic compounds [5]. Cocoa-rich products can have beneficial effects on human health due to their beneficial antioxidant components. For instance, it has been proposed that cocoa products can prevent degenerative diseases, metabolic disorders, and cancer, acting as antiobesogenic, antidiabetic, and antihypertensive factors that are associated mainly with the number of phenolic compounds accounting for approximately 8% of cocoa beans [6,7,8]. The stem bark of cocoa has also shown anti-inflammatory properties [9]. Regarding cocoa bean shells, several revalorizations have been proposed for food, livestock feed, or industrial uses, and several health properties have been reported, including anticarcinogenic, antibacterial, anti-inflammatory, antidiabetic, antiviral, neuroprotective, and cardioprotective effects [5]. There are three main varieties of cocoa cultivated in the Amazon region, with the “forastero” variety being the variety that receives the greatest degree of exploitation. This variety is responsible for 96% of worldwide production due to its resistance to pests [10,11]. The main phenolic compounds in cocoa are the group of flavonoids, which include anthocyanins, flavonols, and flavanols. Other phenolic compounds found in cocoa products are amino acid and phenolic acid conjugates (N phenol amino acids, NPAs), stilbenes, and phenolic acids [12]. Finally, cocoa products contain a good quantity of alkaloids known as methylxanthines [13]. The purine alkaloids theobromine (3,7-dimethylxanthine), caffeine (1,3,7-trimethylxanthine), and theophylline (1,3-dimethylxanthine) are the most common methylxanthines found in cocoa [14]. They are biologically active alkaloids responsible for the bitter taste of cocoa. These alkaloids also possess desirable pharmacological effects, e.g., gastric secretion, diuresis, bronchodilation stimulation of the central nervous system, and stimulation of skeletal muscles in high doses. To date, there is little research conducted about nutritional properties and chemical fingerprinting of the metabolites from cocoa waste products; however, cocoa pod husk is considered a cheap and good source of pectin [2]. In the present study, we present the phenolic composition of two flours made from waste (vein and pod husk) from cocoa CCN-51 variety and their antioxidant potential together with nutritional properties, the content of fatty acids, methylxanthines, proximal composition, and mineral content. Therefore, this study aims to describe the chemical fingerprinting by UHPLC–MS analysis and nutritional properties of cocoa waste products, namely, flours made from vein and pod husk (Figure 1) of a selected Peruvian ecotype of cocoa, evaluation of the antioxidant activity, and their potential as a food or food product.

## 2. Materials and Methods

### 2.1. Chemicals

Ultra-pure water (<5 µg/L TOC) was obtained from the water purification systems Arium 126 61316-RO, plus an Arium 611 UV unit (Sartorius, Goettingen, Germany). Formic acid (MS grade) and methanol (HPLC grade) were purchased from J.T. Baker (Phillipsburg, NJ, USA). Folin–Ciocalteu (FC) reagent, 2,2-diphenyl-1-picrylhydrazyl (DPPH), ferric chloride hexahydrate, 2,4,6-tris(2-pyridyl)-s-triazine, quercetin, gallic acid, Amberlite^®^ resin (XAD4), phosphate buffer, trichloroacetic acid, ferric chloride, hydrochloric acid, ascorbic acid, 2,2′-azino-bis(3-ethylbenzothiazoline-6-sulfonic acid) (ABTS), 2,4,6-tris(2-pyridyl)-s-triazine (TPTZ), gallic acid, potassium hexacyanoferrate(III), 6-hydroxy-2,5,7,8-tetramethylchroman-2-carboxylic acid (Trolox), and dimethylsulfoxide (DMSO) were obtained from Sigma-Aldrich; sodium carbonate, ferrous sulfate, sodium persulfate, sodium acetate, sodium sulfate anhydrous, and ethanol were obtained from Merck (Lima, Peru). HPLC standards (with purity higher than 95% by HPLC) were purchased from Sigma Aldrich Chem. Co. (St. Louis, MO, USA) or Extrasynthèse (Genay, France).

### 2.2. Plant Material

The cocoa fruits (*Theobroma cacao*), CCN-51 variety, were collected in October 2020 directly from the cocoa farm of Mr. Américo Hernández, located in the Pajonal zone, Pardo Miguel district, Rioja province, San Martín region, Peru (05°43’22.5” S and 77°29’20” W, 800 m altitude). The selected fresh cocoa fruits were taken to the laboratory in dark bags where they were washed, brushed, disinfected using a 200-ppm sodium hypochlorite solution for 30 min, and rinsed with water. Then, the pulp was removed, and the fruit opened, wherein the veins were removed, and the pod husks were separated. Then, thermal drying for both waste parts of cocoa fruits was carried out at 70 °C for 48 h. Finally, a blade mill was used (Grindomix GM 200), and the flours were milled using each part of waste and stored. The extract for HPLC analysis and biological activity was prepared by extracting the flour (500 mg), with 10 mL of HPLC-grade methanol with 1% formic acid for 10 min with sonication (3 times). Extractions were combined, the solvent was evaporated under vacuo and filtered thorough Whatman paper number 1, and the yellow gummy residue was obtained (19.5 mg for the vein extract (3.9%) and 12.7 mg for the pod (2.54%) and stored at –20 °C.

### 2.3. HPLC–MS Parameters

A Thermo Scientific Dionex Ultimate 3000 UHPLC machine that was connected by ESI II probe to a Thermo Q exactive spectrometer was used. For the analysis, 5 mg of the flour extract was dissolved in 2 mL of methanol and filtered (PTFE filter), and 10 μL was injected into the instrument. Liquid chromatography was performed using an UHPLC C18 column (Luna© Omega C18 100 Å, Phenomenex (150 mm × 2.1 mm ID, 1.6 µm)) operated at 30 °C. The detection wavelengths were 330, 254, 280, and 354 nm, and PDA from 200 to 800 nm for characterizing the peaks. Mobile phases were 1% formic aqueous solution (A) and 1% formic acid in acetonitrile (B). The gradient used was (0.00 min, 5% B); (1.00 min, 5% B); (25.00 min, 95% B); (26.00 min, 95% B); (30.00, 5% B); and 20 min for column equilibration before each injection. The flow rate was 0.3 mL/min, and the injection volume was 10 μL. Standards and the flour extract dissolved in methanol were kept at 10 °C during storage in the autosampler. The LC–MS and HESI II and Orbitrap spectrometer parameters were optimized as Full MS scan: AGC target: 5 × 10^6^ resolution: 35,000, maximum IT: 80 ms, range: *m/z* 100–1500, microscans: 1, parameters for MS^2^ maximum IT: 100 ms, AGC target: 1 × 10^6^, resolution: 17,500, ionization source parameters: ESI (positive and negative) spray volt: 3.5/2.5 KV, gas heater temp: 280/280 °C, capillary temperature: 260 °C, carrier gas: N_2_ (sheath gas flow rate: 48, sweep gas flow rate: 2, S-lens RF level: 100).

### 2.4. HPLC–DAD Analysis of Catechins and Methylxanthines

Catechins and methylxanthines by HPLC were carried out by means of a chromatographic analysis according to the protocol of Oliviero et al. [15] and Brunetto et al. [16], with some modifications. A chromatograph (Hitachi LaCrhom Elite^®^, Technologies America, Inc., Clarksburg, MD, USA) equipped with a vacuum degasser, a quaternary pump, and a diode-array detector (DAD), calibrated at 280 nm, was employed. The separation of (+)-catechin and (–)-epicatechin was carried out in an RP-18E column whose dimensions were 5 µm in particle size, 250 mm in length, and 4.6 mm in diameter. As mobile phase, methanol (A) acidified with 0.1% formic acid (B) was used, with elution gradients of 0.01 min 60% of A; 5–12 min 80% A; 13–14 min 60% of A. The flow rate of the mobile phase was 1.0 mL/min. The identification of the peaks was carried out by comparing with standards of (+)-catechin and (–)-epicatechin (Sigma-Aldrich^®^, St. Louis, MO, USA) and theobromine and caffeine standards (Sigma-Aldrich^®^, St. Louis, MO, USA). The theobromine and caffeine separation were carried out with the same column as above.

### 2.5. Determination of Proximate Composition

AOAC procedures were used in all determinations [17,18]. The water content was determined by oven-drying the sample up to a constant weight, the crude protein content by the Kjeldahl method (N × 6.25), the fiber content by gravimetric method after acidic hydrolysis of the samples, the total lipid extracted in a Soxhlet apparatus using petroleum ether as solvent, and the ash content by incineration in a muffle furnace at 550 ± 15 °C. Total carbohydrates were calculated as difference: 100 − (g water + g protein + g fiber + g fat + g ash). Results were expressed in grams per 100 *g* fresh weight (g/100 g fw). The experiments were carried out in triplicate.

### 2.6. Mineral Analysis

For the mineral analysis, the flours were dried to ash at 550 °C [18]. The ash in each case was boiled with 10 mL of 20% hydrochloric acid in a beaker, and then filtered into a 100 mL standard flask and made up to 100 mL with distilled deionized water. Levels of minerals, potassium (K), calcium (Ca), magnesium (Mg), sodium (Na), zinc (Zn), manganese (Mn), copper (Cu), and iron (Fe) were determined from the resulting solution using atomic absorption spectroscopy (Varian AA240). The values obtained for each parameter are averages of three determinations for a given food sample.

### 2.7. Fatty Acid Profile

Fats were cold extracted using the Bligh and Dyer method [19]. Fatty acid profiles were obtained by gas chromatography of the fatty acid methyl ester derivatives. Derivatives were obtained by esterification with KOH in methanol (2 M). The fatty acid derivatives were extracted with hexane and analyzed through a Varian-450 gas chromatograph (Varian Inc., Palo Alto, CA, USA). The chromatograph was equipped with a VF-WAXms (60 m × 0.25 mm) capillary column and flame ionization detector (FID). Helium was used as the carrier gas, and the temperature program was as follows: 3 min at 130 °C, gradual heating to 220 °C for 9 min, 35 min at 220 °C, cooling to 130 °C, and 130 °C for 5 min. Individual peaks were identified by referring to a fatty acid methyl ester standard solution and analyzed under the same operation conditions [17].

### 2.8. Antioxidant Activity

#### 2.8.1. DPPH Scavenging Activity

DPPH scavenging activity was determined by the method developed by Brand-Williams et al. [20]. To 3.9 mL of a solution of the DPPH• radical (100 μM) dissolved in 80% methanol, 0.1 mL of the extract (2 mg/mL), previously filtered on a membrane filter (0.45 μm), was added, and the mixture was stirred vigorously and set in the dark for 30 min at 25 °C. After that time, the absorbance at 517 nm was read in a UV-visible Cary60 spectrophotometer. The concentration of DPPH• in the reaction medium was obtained from a calibration curve by linear regression. The control consisted of 0.1 mL of 80% aqueous methanol and 3.9 mL of DPPH • solution (100 µM). The results are expressed in TEAC, that is, antioxidant activity equivalent to Trolox (μmol Trolox/g of extract). The reference synthetic antioxidant Trolox, at a concentration of 5–30 µM in 80% methanol solution, was tested under the same conditions.

#### 2.8.2. ABTS Bleaching Capacity

ABTS bleaching capacity was determined by the method developed by Re et al. [21]. The reaction was started with the addition of 1500 μL of an ABTS•+ solution in PBS buffer (0.70 ± 0.02 at λ = 734 nm) to 500 μL of the extract (2 mg/mL) in a cuvette kept at 30 °C. It was homogenized and allowed to react for 7 min, and then the absorbance reading was made at a wavelength of 734 nm, using a Cary60 UV-visible spectrophotometer. The results are expressed in TEAC (μmol Trolox/g of extract). The calibration curve for TEAC was constructed using different concentrations of Trolox (4–14 µM) in PBS buffer solution under the same conditions.

#### 2.8.3. Ferric-Reducing Antioxidant Power Assay (FRAP)

The ferric-reducing antioxidant power (FRAP) was determined according to the Benzie and Strain method [22]. A volume of 10 μL of the extract (2 mg/mL) was mixed with 90 μL of distilled water and 900 μL of the FRAP reagent (2.5 mL of the 2,4,6-tripyridyl-s-triazine solution at a concentration of 10 μM in HCl 40 mM; 2.5 mL of FeCl_3_ 20 μM and 25 mL of acetate buffer 0.3 μM at a pH of 3.6). The absorbancy was read at 593 nm after 7 min in a UV-visible Cary60 spectrophotometer. The results are expressed in TEAC, that is, antioxidant activity equivalent to Trolox (μmol Trolox/g of extract).

#### 2.8.4. Total Phenolic (TP) Content

The content of total phenols was estimated by a colorimetric method based on the procedures described by Velioglu et al. [23] with some modifications. In essence, 100 µL of the extract (2 mg/mL) was mixed with 750 µL of the Folin–Ciocalteu reagent diluted in a 1/10 proportion of Milli-Q water. After 5 min in the dark, 750 µL of sodium bicarbonate (60 g/L) was added to the mixture. The tubes were kept in the dark for 90 min at 30 °C, then the absorbance was read at 725 nm, using a Cary60 UV-visible spectrophotometer. Gallic acid (10–100 μg) was used for the construction of the standard curve. The results are expressed as milligrams of gallic acid/g extract.

### 2.9. Statistical Analysis

All the experiments were repeated at least three times. The results were expressed as mean ± standard deviation (SD) using GraphPad Prism 8. Comparison of results was performed using one-way analysis of variance (ANOVA), followed by Tukey’s HSD (honest significant difference) test (*p* < 0.05).

## 3. Results and Discussion

### 3.1. UHPLC–MS Analysis of Cocoa Extract

**Figure 2:**
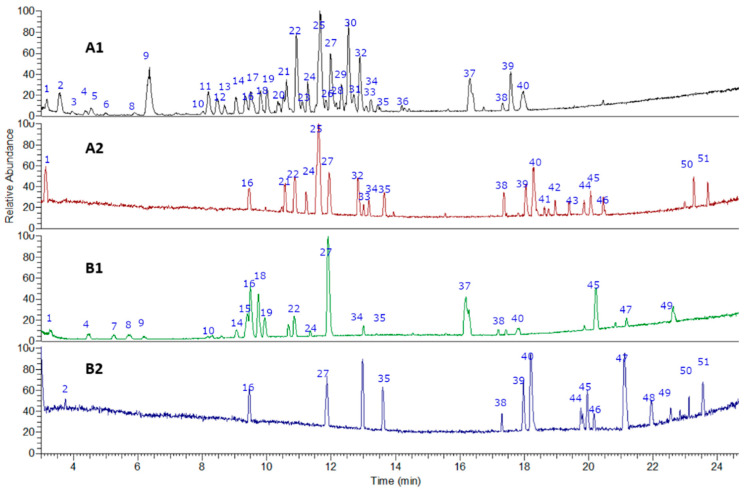
UHPLC–PDA–ESI–OT–MS/MS chromatograms (TIC, total ion current) of (**A1**) pod husk flour negative mode, (**A2**) pod husk flour positive mode, (**B1**) vein flour negative mode, and (**B2**) vein flour positive mode.

**Figure 3:**
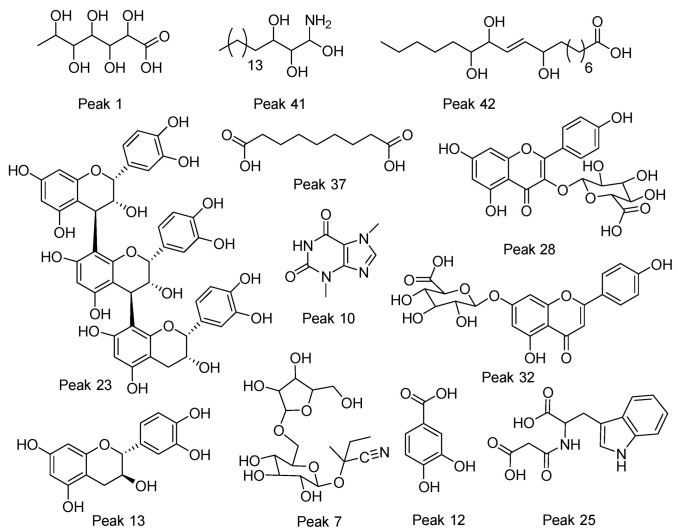
Structures of some representative compounds in vein and pod husk flours of cocoa.

The fingerprinting of the flour from cocoa pod husk and vein were created and investigated by means of UHPLC–high-resolution MS with DAD analysis. The negative mode was used for the identification of phenolic compounds, while the positive mode was used for anthocyanins and methylxanthines. Some of the metabolites identified are reported for the first time in flours made from waste from this species. In total, 51 metabolites were detected and tentatively identified including phenolic acids, amino acids, anthocyanins, flavonoids, alkaloids, terpenes, and fatty acids (See Figure 2 and Figure 3 and Table 1). A detailed analysis is provided below.

#### 3.1.1. Saturated Organic Acids

Peak 1 was identified as gluconic acid (C_6_H_12_O_7_), peak 2 as tartaric acid (C_4_H_6_O_6_), peak 5 as succinic acid (C_4_H_6_O_4_), peak 9 as 2-isopropylmalic acid (C_7_H_12_O_5_), peak 11 as citrate (C_6_H_8_O_7_), and peak 7 as 6’-apiosyllotaustralin (C_16_H_27_NO_10_). Peaks 15 and 17 were identified as isomers of everlastoside C (C_16_H_30_O_10_).

#### 3.1.2. Fatty Acids or Derivatives

Peak 37 was identified as the dicarboxylic azelaic acid (C_9_H_16_O_4_), peaks 30 and 40 as the poliols heptadecasphinganine isomers, peak 41 as phytosphingosine, and peaks 42 and 45 as the isomers trihydroxyoctadecenoic acid (*m/z* 329.2336, C_18_H_34_O_5_). Peaks 46–51 were identified as stearic acid, oleic acid, araquidonic acid, linolenic acid, linoleic acid, and margaric acid, respectively. Their quantities are depicted in Table 2.

#### 3.1.3. Procyanidins

Peak 27 was identified as procyanidin type B isomer 1 (C_30_H_26_O_12_), peak 13 as catechin, peak 20 as (-)-epicatechin (C_15_H_14_O_6_), peak 23 as procyanidin C1 (C_45_H_38_O_18_), and peak 35 as procyanidin type B (C_30_H_26_O_12_).

#### 3.1.4. Flavonols

Peak 34 with a M-H ion at *m/z* 463.0863 and diagnostic MS ions at *m/z* 300.0276, 178.9982, and 161.0450 was identified as quercetin 3-O-glucoside (C_21_H_20_O_12_); peak 29 as quercetin 3-*O*-arabinose (C_20_H_18_O_11_); peak 32 as apigenin-7-*O*-glucuronide (C_21_H_18_O_11_); and peak 19 with a M-H ion at *m/z* 593.1520 and diagnostic c-glycosyl fragments at 503.1200, 473.1094, 383.0772, and 353.0670 as 6,8-C-dihexosylapigenin (C_27_H_30_O_15_). The same occurred with isomer compound peaks 21 and 22, 8-C-Glucosyl-6-C-arabinosylapigenin and 6-C-Glucosyl-8-C-arabinosylapigenin, respectively (C_26_H_28_O_14_). Peaks 28 and 33 were devoided as kaempferol 3-glucuronide (C_27_H_18_O_12_) and kaempferol 3-*O*-pentoside (C_20_H_18_O_10_), respectively.

#### 3.1.5. Phenolic Acids

Peak 3 with a M-H ion at *m/z* 329.0883 and diagnostic ions at *m/z* 167.0345, 152.0109, and 123.0444 was identified as vanillic acid 4-hexoside (C_14_H_18_O_9_); in the same manner, peak 4 as 1-O-syringoyl-glucopyranose (C_15_H_20_O_10_), peak 12 as protocatechuic acid (C_7_H_6_O_4_), peak 14 as benzyl O-[pentosyl-hexoside] (C_18_H_26_O_10_), and peak 36 as gentisic acid 5-O-hexoside (C_13_H_16_O_9_) showing ions at 165.0187, 152.0109, 108.0208, and 85.0285.

#### 3.1.6. Amino Acids

Peak 18 was identified as the amino acid derivative methoxytyrosine (C_10_H_13_NO_4_); in the same way, peaks 25 and 26 were regarded as malonyltryptophan (C_14_H_14_N_2_O_5_) and N-acetyltryptophan (C_13_H_14_N_2_O_3_), respectively.

#### 3.1.7. Alkaloids

Peaks 10 and 16 were identified as theobromine (C_7_H_8_N_4_O_2_) and caffeine (C_8_H_10_N_4_O_2_), respectively.

### 3.2. Chemical Composition and Nutritional Properties of Flours

The chemical composition and nutritional properties of flours were performed according to previous methodologies [15,16,17,19]. This included proximal analysis (Table 2), mineral content (Table 3), and content of fatty acids (Table 4). Table 2 shows proximal composition such as the humidity, ashes, protein, lipids, carbohydrates, and fiber, while Table 3 shows the mineral contents of two flours for cocoa waste material. The results of physicochemical properties showed that the proximal composition and the caloric value of these flours made of cocoa waste had a high fiber content that is good for use as a supplement (35.48 ± 1.47 and 7.26 ± 0.17% in pod husk and vein flour, respectively) and carbohydrate content (41.89 and 57.96%, respectively), which make them two highly caloric flours. The composition showed differences with other flours derived from foods, such as banana flour (*Musa paradisiacal* L.), which showed total starch of 73.36% and dietary fiber of 14.52% [24], while in buckwheat, for instance, in the flour, these values were dietary fiber: 6.77%, ashes: 1.82%, starch: about 78.4%, protein: 10%, and lipid content: 2%, but in its bran, starch: 40.7%, ashes: 4%, protein: above 21%, and lipid content: around 7% [25]. Soy flours collected at Cedar Rapids showed protein at 52%, fat at 0.8%, and ashes at 6.31% [26], while a group of flours obtained from the species of *Prosopis* showed protein: 7.17–11.2%, crude fiber: 2.7–3.4%, and carbohydrates: all around 8.2% [27]. The composition of cocoa pod husk in the literature showed great variability; however, our results are in accordance with those reported by Mariatti et al. [28]. Briefly, ashes: 5.9–13.0 (8.62%), lipids: 0.6–4.7 (0.51%), proteins: 2.9–9.1 (8.50%), fiber: 18.3–59.0 (35.48%), and carbohydrates: 17.4–47.0 (41.89%) (Table 2). The flours were analyzed for mineral content (Ca, Na, K, Mg, Cu, Mn, Zn, and Fe) and were high in K (112.04 and 54.03 mg K/100 g for pod husk and vein, respectively) and low in sodium (0.47 and 0.09 mg Na/100 g for pod husk and vein, respectively), which is also good for hypertensive people. The pod husk had the highest contents of Mg, Ca, Mn, Zn, and Fe (Table 3). The content of Ca makes the flour a good supplement for bones. The cocoa pod husks have been studied for use as fertilizers due to their high mineral content [2]. The fatty acid profile of the flours was investigated using a standardized protocol [19]. Saturated fatty acids were 44.99% and 39.40% in vein and pod husk flours, respectively (Table 4). While the pod husk showed more polyunsaturated fatty acids (52.54%), the vein showed more monounsaturated fatty acids (37.11%), and thus the pod husk flour has more healthy fatty acids. Indeed, the pod husk is richest in dietary poly-UFAs, and it is more suitable as a food or food supplement for its nutritional values. Because of its high values in proteins, crude fats, fibers, and mineral levels, cocoa pod husk has been widely explored as feed for poultry and/or animals [3]. Moreover, the main methylxanthine alkaloid compounds: theobromine, caffeine, and the two main catechins were quantified, and the results are shown in Table 5. The pod husk showed the highest content of theobromine (10.21 µg/g), while the vein showed the highest content of caffeine, epicatechin, and catechin (1.11 µg/g, 3.40 µg/g, and 3.09 mg/g, respectively; Table 5).

### 3.3. Antioxidant Activity and Total Polyphenol Content

The antioxidant capacity cannot be fully described by only one method; thus, for this study, we employed three different complementary antioxidant methods (ABTS, DPPH, and FRAP) that have been applied to the flours in addition to the phenolic content measured by spectrophotometry. It has been reported that the total phenolic content of cocoa varies on the basis of the growing region and extraction solvent technique [29]. Table 6 shows the antioxidant capacities of the three methods used in this study. The DPPH antiradical activity as well as the reducing power of the flours can serve as a significant indicator of their potential antioxidant activity. For this reason, the reducing power of ferric ions and total polyphenol content was also examined in the two cocoa waste flours (Table 6). The FRAP of the flour from the vein exhibited a weak reducing power compared to the pod husk, but the total phenolic content (111.05 mg GAE/g flour) was higher than those reported for cocoa pod husk flour (CPHF) criollo variety (5.4 to 16.6 mg GAE/g flour) [30]. This difference may have been due to the location growth of cocoa, variety, and the solvent system used in the extraction of phenolics [29]. To show some comparison, antioxidant properties of refined and whole wheat flour by the oxygen radical absorbance capacity (ORAC) test for refined wheat flours ranged from 10.88 to 14.38 µmol TE/g (mean 12.52 µmol TE/g) while showing significantly lower values compared to their whole wheat flour counterparts, which ranged from 27.93 to 44.33 µmol TE/g (mean 35.74 µmol TE/g) from the same brand [31]. In addition the DPPH content reported for those wheat flours ranged among 4–5 μmol equivalent of Trolox/g [31], which is lower than our cocoa waste flours (46 and 87 μmol equivalent of Trolox/g for the vein and husk flours, respectively) (Table 6).

Furthermore, our values for the TPC (Table 6) were higher than those reported for the TPC of cocoa shell hydroalcoholic extract (51.9 mg/g) [32] and lower than those obtained by Amin et al. (113 mg/g extract) who used ethanol as an extraction solvent [33]. Our DPPH activity was also higher (Table 6) than that reported by Grillo et al. (83.1 ± 5.3 mg/mL) [32] and is in accordance with that reported by Delgado-Ospina et al. (36 to 133 µmol Trolox/g) [30]. Furthermore, strong correlation was found between total phenolic and the three antioxidant assays DPPH (r = 0.9995, *p* < 0.001), ABTS (r = 0.9970, *p* < 0.0001), and FRAP (r = 0.9988, *p* < 0.0001).

In the flours, the antioxidant compound protocatechuic acid, PCA (3,4-dihydroxy benzoic acid), was detected; this is one of the main metabolites produced by anthocyanins and proanthocyanins and has been shown to possess antioxidant activity in vitro and in vivo [34]. In addition, catechin, epicatechin, and procyanidins detected in the flours are present in chocolate made of cocoa and considered the main phenolics in it (30%), being directly related to its antioxidant capacity [35]. The flavanols detected in cocoa food products (such as apigenin-7-*O*-glucuronide, quercetin 3-O-glucoside, and kaempferol 3-*O*-pentoside) are also responsible for the antioxidant activity of those products [36]. On the other hand, dietary-important unsaturated and saturated fatty acids were also detected, and saturated ones were measured by GC-FID (Table 4). In the cocoa flours, it was shown that chocolates showed a high concentration of saturated fatty acids, mainly stearic acid (18:0), and palmitic acid (16:0), followed by the unsaturated fatty acids, among which linoleic acid (18:6) and oleic acid (18:1n-9) were the more concentrated [37], having been very important in stopping the development of coronary diseases and high blood pressure [38].

## 4. Conclusions

In this study, two flours made of the vein and pod husk from a special ecotype of Peruvian cocoa (CCN-51) were investigated regarding their potential as a food or food supplement and their antioxidant capacities. Different compounds were detected in the two flours including methylxanthines, catechin, flavonoids, fatty acids, amino acids, phenolic acids, and other common acids. The principal methylxanthines and catechins were quantified, and the content of fatty acids was quantified individually for each waste material, making it a standardized food waste material. Proximal composition and mineral content were analyzed for the first time in these by-products, and the findings of physicochemical characteristics revealed that these flours generated from cocoa waste had a significant fiber and carbohydrate content, making it an energetic and healthy product. The mineral content (Ca, Na, K, Mg, Cu, Mn, Zn, and Fe) of these flours is shown to be a rich source of potassium and low in sodium, which is good for people suffering from high blood pressure. The pod husk had the highest contents of main dietary minerals, and therefore is better for this purpose. Flours made of cocoa waste have good nutritional properties and can be a good source of dietary phenolic compounds, which are essential for the preparation of nutraceuticals or food supplements. More biological tests and more analyses are necessary to test the health potential of these waste flours.

## Figures and Tables

**Table 1:** High resolution UHPLC–PDA-Q orbitrap identification of metabolites in vein and pod husk flours of cocoa.

Peak #	Rt	MS–ESI^−^ or MS–ESI^+^	MSn	Error (ppm)	Molecular Formula	Tentative Identification
1	1.62	195.0507	195.0507, 159.0293, 129.0186, 75.0078	4.16	C_6_H_12_O_7_	Gluconic acid
2	1.70	149.0086	130.9978, 103.0028, 87.0078, 72.9921	3.33	C_4_H_6_O_6_	Tartaric acid
3	3.57	329.0883	167.0345, 152.0109, 123.0444	3.41	C_14_H_18_O_9_	Vanillic acid 4-hexoside
4	4.38	359.0989	197.0453, 182.0215, 153.0551, 138.0315	3.12	C_15_H_20_O_10_	1-O-Syringoyl-glucopyranose
5	4.41	117.0186	99.0986, 73.0285	2.78	C_4_H_6_O_4_	Succinic acid *
6	4.54	367.1614	235.1187, 161.0450, 143.0348, 113.0235, 101.0235, 71.0128	2.61	C_17_H_28_O_10_	Unknown
7	5.24	438.1624	392.1567, 293.0884, 233.0671, 191.0560, 161.0449, 125.0237, 89.0235	4.13	C_16_H_27_NO_10_	6’-Apiosyllotaustralin
8	5.73	433.1930	281.1397, 219.1388, 161.0452, 119.0342101.0235, 89.0235	4.17	C_21_H_31_O_10_	Unknown
9	6.21	175.0609	157.0502, 146.9608, 131.0707, 115.0392, 113.0600, 85.0649	4.46	C_7_H_12_O_5_	2-Isopropylmalic acid
10	8.02	181.07197	67.0282	2.83	C_7_H_8_N_4_O_2_	Theobromine *
11	8.32	191.0194	173.0087, 129.0186, 111.0079, 87.0078, 85.0285	3.98	C_6_H_8_O_7_	Citrate
12	8.46	153.0188	109.0287	0.30	C_7_H_6_O_4_	Protocatechuic acid *
13	8.72	289.0722	245.0821, 205.0506, 203.0711, 179.0342, 165.0180, 151.0392, 125.0233	2.79	C_15_H_14_O_6_	(+)-Catechin *
14	8.84	447.1513 [M+FA-H]	401.1456, 269.1032, 233.0673, 161.0449, 113.0236, 101.0235	2.37	C_18_H_26_O_10_	Benzyl O-pentosyl-hexoside
15	9.05	427.1826 [M+FA-H]	381.1770, 249.1345, 161.0449, 101.0235, 71.0129	2.57	C_16_H_30_O_10_	Everlastoside C
16	9.35	195.08832	195.0832	1.45	C_8_H_10_N_4_O_2_	Caffeine *
17	9.50	427.1824 [M+FA-H]	381.1768, 249.1344, 161.0450, 101.0235, 71.0128	1.99	C_16_H_30_O_10_	Everlastoside C isomer
18	9.59	210.0770	210.0769, 124.0396, 94.0288	1.89	C_10_H_13_NO_4_	Methoxytyrosine
19	9.90	593.1520	503.1200, 473.1094, 383.0772, 353.0670	2.25	C_27_H_30_O_15_	6,8-C-Dihexosylapigenin
20	10.01	289.0720	245.0820, 205.0504, 203.0710, 179.0345, 165.0188, 151.0394, 125.0236	2.79	C_15_H_14_O_6_	(-)-Epicatechin *
21	10.41	563.1413 565.1548	545.1313, 503.1196, 473.1091, 443.0986, 425.0883, 383.0777, 353.0369, 529.1335, 511.1228, 481.1122, 427.1020, 409.0912, 379.0805, 325.0700	2.27 –1.56	C_26_H_28_O_14_	8-C-Glucosyl-6-C-arabinosylapigenin
22	10.61	563.1412 565.1548	545.1301, 503.1197, 473.1091, 443.0986, 425.0880, 383.0773, 353.0669, 529.1330, 511.1226, 481.1121, 427.1018, 409.0911, 379.0805, 325.0699	2.06 –1.56	C_26_H_28_O_14_	6-C-Glucosyl-8-C-arabinosylapigenin
23	10.52	865.1994	695.1393, 577.1351, 407.0774, 289.0720, 243.0398, 161.0283, 125.0236	1.63	C_45_H_38_O_18_	Procyanidin C1
24	10.91	563.1414 565.1075	545.1304, 503.1186, 473.1091, 443.0986, 425.0896, 529.1330, 511.1226, 481.1121, 427.1018, 409.0911	2.27 –0.82	C_26_H_28_O_14_	8-C-Galactosyl-6-C-arabinosylapigenin
25	11.90	289.0832 291.0973	245.0934, 203.0823, 135.1548, 116.0344, 98.0238, 74.0236, 273.0863, 245.0917, 227.0810, 201.1018, 188.0703, 159.0914, 130.0650	4.37 –0.99	C_14_H_14_N_2_O_5_	Malonyltryptophan
26	11.90	245.0932	203.0822, 160.0246, 116.0344, 98.0238, 74.0237, 70.0288	4.49	C_13_H_14_N_2_O_3_	N-Acetyltryptophan
27	11.91	577.1361 579.1495	407.0735, 289.0720, 245.0820,161.0238, 125.0236, 409.0910, 287.0544, 275.0544	2.62 –1.33	C_30_H_26_O_12_	Procyanidin type B isomer 1
28	11.99	461.0730	285.0406, 113.0285, 85.0285	2.10	C_27_H_18_O_12_	Kaempferol 3-glucuronide
29	12.45	433.0781	300.0278, 178.9981, 151.0028	2.41	C_20_H_18_O_11_	Quercetin 3-*O*-Arabinose
30	12.53	439.1073	409.0967, 260.0360, 110.9749	–2.05	C_16_H_24_O_14_	Unknown
31	12.53	439.1071	409.0964, 260.0364, 110.9748, 96.9591	–2.53	C_16_H_24_O_14_	Unknown
32	12.89	445.0779	269.0456, 175.0242, 113.0235, 85.0284	1.85	C_21_H_18_O_11_	Apigenin-7-*O*-glucuronide
33	12.99	417.0830	284.0327, 255.0297, 209.0455, 151.0030	1.94	C_20_H_18_O_10_	Kaempferol 3-*O*-pentoside
34	13.00	463.0863	300.0276, 178.9982, 161.0450	2.03	C_21_H_20_O_12_	Quercetin 3-O-glucoside
35	9.43	577.1361 579.1495	407.0775, 289.0720, 245.0821, 161.0238, 125.0236, 409.0910, 287.0544, 275.0544, 247.0597, 163.0387, 127.0389	3.46 –1.33	C_30_H_26_O_12_	Procyanidin type B
36	13.26	315.0727	165.0187, 152.0109, 108.0208, 85.0285	5.18	C_13_H_16_O_9_	Gentisic acid 5-O-hexoside
37	13.10	187.0971	169.0864, 143.1071, 125.0963		C_9_H_16_O_4_	Azelaic acid
38	17.25	373.1870	331.1780, 177.0552, 165.0550, 59.0128	3.46	C_18_H_30_O_8_	Unknown
39	18.04	288.2894	106.0865, 88.0861	0.96	C_17_H_37_NO_2_	Heptadecasphinganine isomer 1
40	18.29	288.2895	270.2783, 106.0864, 88.0760	–0.85	C_17_H_37_NO_2_	Heptadecasphinganine isomer 2
41	19.07	318.2999	300.2891, 282.2785, 270.2785, 252.2680, 60.0450	–1.07	C_18_H_39_NO_3_	Phytosphingosine
42	19.30	329.2335	229.1443, 211.1336, 171.1020, 139.1120	3.67	C_18_H_34_O_5_	9,12,13-Trihydroxyoctadecenoic acid
43		493.2294 [M+FA-H]	447.2236, 315.1813, 161.0449, 143.0340, 113.0235, 101.0234, 71.0128	3.00	C_21_H_36_O_10_	Unknown
44	17.35	274.2737	256.2629, 106.0864, 88.0761	–1.26	C_16_H_37_NO_2_	Unknown
45	17.96	329.2336	311.2228, 201.1127, 171.1020, 129.0917	4.04	C_18_H_34_O_5_	Trihydroxyoctadecenoic acid isomer 3
46	19.87	285.24893	155.11879		C_18_H_33_O_2_	Stearic acid *
47	22.63	281.24887	152.99487	4.85	C_18_H_34_O_2_	Oleic acid *
48	23.39	303.23272, 305.23784	259.2525, 231.2116		C_20_H_32_O_2_	Araquidonic acid *
49	21.36	277.21603	220.11197, 181.01431	–0.62	C_18_H_30_O_2_	Linolenic acid *
50		279.23186, 281.23198	259.2436, 231.2112	4.31	C_18_H_32_O_2_	Linoleic acid *
51		269.24899, 271.24878	143.03407	5.51	C_17_H_34_O_2_	Margaric acid

* Determined by spiking experiments using authentic standards. # means number

**Table 2:** Proximal composition in flour from vein and pod husk of cocoa (%).

Cocoa Waste	Humidity	Ashes	Total Lipids	Crude Protein	Crude Fiber	Carbohydrates
Vein flour	15.2 ± 0.10 ^a^	6.42 ± 0.26 ^a^	0.42 ± 0.02 ^a^	12.74 ± 0.01 ^a^	7.26 ± 0.17 ^a^	57.96
Pod husk flour	5.00 ± 0.06 ^b^	8.62 ± 0.07 ^b^	0.51 ± 0.02 ^b^	8.50 ± 0.00 ^b^	35.48 ± 1.47 ^b^	41.89

Each value represents the means ± SD of three replicates, *n* = 3, while different letters on the same column indicate significance difference using Tukey test at 0.05 level of significance (*p* < 0.05).

**Table 3:** Mineral content (mg/100 g) in flour from vein and pod husk of *cocoa*.

Cocoa Waste	Fe	Zn	Mn	Cu	Mg	K	Na	Ca
Vein flour	4.49 ± 0.18 ^a^	2.97 ± 0.04 ^a^	1.02 ± 0.02 ^a^	0.76 ± 0.03 ^a^	7.60 ± 0.05 ^a^	54.03 ± 0.11 ^a^	0.09 ± 0.00 ^a^	1.38 ± 0.02 ^a^
Pod husk flour	5.04 ± 0.10 ^b^	7.22 ± 0.12 ^b^	7.32 ± 0.12 ^b^	8.55 ± 0.28 ^b^	21.23 ± 0.22 ^b^	112.04 ± 0.18 ^b^	0.47 ± 0.02 ^b^	6.15 ± 0.04 ^b^

Each value represents the means ± SD of three replicates, *n* = 3, while different letters on the same column indicate significant difference using Tukey test at 0.05 level of significance (*p* < 0.05).

**Table 4:** Fatty acids profile in flour from vein and pod husk of cocoa (%).

Fatty Acid Profile	Cocoa Waste	
	*Vein Flour*	*Pod Husk Flour*
c12:0 (lauric acid)	0.685 ± 0.01 ^a^	0.564 ± 0.01 ^b^
c14:0 (miristic acid)	0.634 ± 0.02 ^a^	1.366 ± 0.01 ^b^
c16:0 (palmitic acid)	26.820 ± 0.11 ^a^	34.324 ± 0.11 ^b^
c16:1 (palmitoleic acid)	0.291 ± 0.01 ^a^	0.430 ± 0.00 ^b^
c17:0 (margaric acid)	0.374 ± 0.01	-
c18:0 (stearic acid)	15.784 ± 0.40 ^a^	3.143 ± 0.03 ^b^
c18:1 (oleic acid)	36.822 ± 0.56 ^a^	7.635 ± 0.12 ^b^
c18:2 (linoleic acid)	15.690 ± 0.19 ^a^	48.924 ± 0.06 ^b^
c18:3 (linolenic acid)	2.212 ± 0.04 ^a^	3.614 ± 0.03 ^b^
c20:0 (arachidonic acid)	0.688 ± 0.05	-
Saturated FAs	44.99%	39.40%
Mono-UFAs	37.11%	8.07%
Poly-UFAs	17.90%	52.54%

Each value represents the means ± SD of three replicates, *n* = 3, while different letters on the same file indicate significant difference using Tukey’s test at 0.05 level of significance (*p* < 0.05).

**Table 5:** Main compound quantitative analyses by HPLC in flour from vein and pod husk of cocoa.

Cocoa Waste	Theobromine (µg/g)	Caffeine (µg/g)	Catechin (mg/g)	Epicatechin (µg/g)
Vein flour	6.32 ± 0.07 ^a^	1.11 ± 0.04 ^a^	3.09 ± 0.15 ^a^	3.40 ± 0.16 ^a^
Pod husk flour	10.21 ± 0.18 ^b^	0.22 ± 0.00 ^b^	2.93 ± 0.15 ^a^	1.14 ± 0.02 ^b^

Each value represents the means ± SD of three replicates, *n* = 3, while different letters on the same column indicate significant difference using Tukey’s test at 0.05 level of significance (*p* < 0.05).

**Table 6:** Antioxidant activity of vein and pod husk flours of cocoa.

Cocoa Waste	DPPH (µmol Trolox/g)	ABTS (µmol Trolox/g)	FRAP (µmol Trolox/g)	Total Phenolics (mg AG/g)
Vein fluor	46.51 ± 1.13 ^a^	104.07 ± 4.25 ^a^	73.77 ± 1.59 ^a^	86.90 ± 0.81 ^a^
Pod husk flour	87.42 ± 1.22 ^b^	155.38 ± 2.96 ^b^	127.44 ± 3.86 ^b^	111.05 ± 1.34 ^b^

Each value represents the means ± SD of three replicates, *n* = 3, while different letters on the same column indicate significant difference using Tukey’s test at 0.05 level of significance (*p* < 0.05).

## Data Availability

The data presented in this study are available in this article. Raw HPLC data or other data can be available on the authors’ request.
